# Identifying diagnostic testing gaps for antiphospholipid syndrome within a single healthcare system

**DOI:** 10.1371/journal.pone.0359712

**Published:** 2026-10-16

**Authors:** Deborah J. Novak, Michelle Grant, Ann Marie Tice, Veena Joy, A. Dhanya Mackeen, Donna M. Wolk

**Affiliations:** 1 Geisinger Medical Laboratories, Geisinger, Danville, Pennsylvania, United States of America; 2 Thermofisher Scientific, Philadelphia, Pennsylvania, United States of America; 3 Geisinger Commonwealth School of Medicine, Scranton, Pennsylvania, United States of America; 4 Obstetrics and Gynecology, Maternal and Fetal Medicine, Geisinger, Danville, Pennsylvania, United States of America; University of Diyala College of Medicine, IRAQ

## Abstract

Antiphospholipid antibody syndrome (APS) is a rare disease with significant morbidity and the possibility of mortality. While there are no established diagnostic criteria for use in clinical settings, there is a large body of global research organized by the American College of Rheumatology and European Alliance of Associations for Rheumatology (ACR/EULAR) into accepted research guidelines, developed for identifying probable APS cases. The research guidelines were used in this study as a surrogate approach for evaluating diagnostic testing patterns, with the aim of identifying potential gaps in patient evaluation for APS in clinical settings. For this study, suggested testing for APS characterization included IgG (Immunoglobulin G) and IgM (Immunoglobulin M) anti-cardiolipin antibodies; IgG and IgM anti-beta-2 glycoprotein I antibodies; and Lupus Anticoagulant. This retrospective cross-sectional study identified patients from the electronic medical records of a regional health system with International Classification of Diseases (ICD) diagnostic codes for APS-associated conditions, signs, symptoms, and/or testing collected for antiphospholipid antibodies in selected medical and surgical specialty locations, from 01/01/2011 to 12/31/2020. These data were used to assess how closely the clinical testing patterns followed the 2023 ACR/EULAR criteria for laboratory evaluation. The data showed that 80.70% of 16679 patients with antiphospholipid antibody tests collected did not include all antiphospholipid antibody testing described in 2023 ACR/EULAR research criteria guidelines. These findings may represent common suboptimal ordering practices in evaluations for APS, highlighting the need for peer comparison and the development of future clinical laboratory guidance to support more complete assessment for APS.

## Introduction

Antiphospholipid antibody syndrome (APS) is a rare systemic autoimmune disease. The incidence of APS is approximately 1–2 cases per 100,000, with a prevalence of 40–50 persons per 100,000 [1,2]. The sex ratio is dissimilar across studies [1]. The pathophysiology of APS involves the development of venous, arterial, and/or micro-thrombosis with manifestations such as deep venous thrombosis, pulmonary embolism, stroke, pregnancy loss, preeclampsia, placental insufficiency, or fetal death. APS may also manifest with certain non-thrombotic signs and symptoms, such as cardiac valve vegetations, thrombocytopenia, and neurological symptoms [3–6].

Timely diagnosis of APS could facilitate initiation of appropriate disease management; however, due to the rarity of the disease and the complexity of testing, clinicians may have incomplete knowledge of current assessment strategies. A 1998 post-Eighth International Congress on APS workshop in Sapporo, Japan, produced standardized classification criteria for characterization of APS for research purposes, which include clinical and laboratory criteria. Clinical laboratory tests included in the criteria were: 1) Lupus Anticoagulant (LA), 2) IgG and IgM anti-cardiolipin antibodies (aCL), and 3) IgG and IgM anti-beta2 glycoprotein I antibodies (aB2GPI). At the 2005 preconference workshop to the Eleventh International Congress on Antiphospholipid Antibodies in Sydney, Australia, modifications were made to the Sapporo Classification criteria based on new research and clinical data [7]. The American College of Rheumatology and the European Alliance of Associations for Rheumatology (ACR/EULAR) Joint Conference met in 2023 to create new research classification criteria aimed at improving the specificity of the previous APS criteria [3,8]. The specific laboratory tests in the previous criteria did not change at the 2023 Joint Conference.

The ACR/EULAR-defined research criteria are available and can be used as guidance in identifying APS in clinical settings. The 2023 ACR/EULAR effort added clinical signs/symptoms entry criteria (which would facilitate pre-test probability) for initial patient assessment, categorizing the various manifestations of the disease into “domains” of disease: macrovascular venous, macrovascular arterial, microvascular (suspected and established), obstetrical, cardiac valvular, and hematological. Interpretative algorithms are also provided in the document [3]. Despite improvements in research definitions used to identify homogeneous APS cohorts for research, there are still no accepted diagnostic criteria for APS for use in the clinical setting.

From a diagnostic laboratory testing perspective, the positivity pattern of test results regarding isotype associations, single-, double-, and triple-positivity results, is thought to aid in the risk assessment for APS [9,10]; and is assembled from the results of all five antiphospholipid antibody tests. There was no change to the requirement of confirmation of positive results (proof of persistence) for interpretation of a positive test: to prove persistence of the positive result, the interpretation of initial APS test results requires confirmatory (repeat) testing for any positive test(s) after at least 12 weeks from initial testing [3,11,12]. Proof of antibody persistence is necessary because conditions such as acute infection or inflammation may induce the transient appearance of antiphospholipid antibodies. This study does not include a review of confirmatory testing. This study’s purpose was to characterize APS-related test ordering patterns in the Geisinger system to demonstrate existing APS test ordering practices and compare concordance with testing as described in 2023 ACR/EULAR guidelines (and ISTH-SSC (Scientific and Standardization Committee of the International Society on Thrombosis and Haemostasis [11,12]. Although the timeline for the data extracted for this study was before the latest 2023 ACR/EULAR work, the testing patterns were retrospectively applied and are identical for this study time period and current guidelines. While this study does not include chart review of confirmatory testing, failure to obtain LA, aCL antibody, and aB2GPI antibody tests in an evaluation for APS could result in a missed/delayed diagnosis [13] and could represent an opportunity for future improvement.

## Materials and methods

This retrospective cross-sectional study aimed to characterize test ordering patterns to assess concordance with 2023 ACR/EULAR APS testing guidelines. Confirmatory patterns are not discussed in this effort, as characterization required chart review, which was unavailable. Data used to describe test ordering patterns were obtained by extracting and reviewing electronic health record data (EPIC EHR) for APS-specific ICD-9 and ICD-10 diagnostic codes and/or APS-associated “test collected” data from the dates 01/01/2011 through 12/31/2020 at Geisinger, an integrated healthcare delivery system in Pennsylvania. No inpatient data were collected because the inclusion criteria specified patients in outpatient clinic locations and Emergency Departments (EDs). Diagnostic codes are provided in the S1 Table – Listing of Diagnostic Codes used to query the Electronic Health System to identify participants who may be subject to testing for antiphospholipid antibody syndrome (APS). Mapping outpatient diagnostic codes was performed using ICD-9 and ICD-10 codes for signs and symptoms potentially associated with APS, and using Epic software functionality created by a Geisinger subject matter group related to coagulation and similar disorders. The data extraction process from the EHR occurred between March 18, 2022, and October 18, 2022. Anonymized data is available on Zenodo doi 10.5281/zenodo.21877037.

Protected Health Information (PHI) was visible to author AMT (a Geisinger “honest data broker”) during data extraction.

In this study, “test collected” was used as a proxy for “test ordered”, to avoid testing that was ordered but not performed. The definition of complete test ordering practices used in the study was the presence of “test collected” data for LA; IgG and IgM aCL antibodies: and IgG and IgM aB2GPI antibodies. The time period defined for complete testing was 12 weeks from the first test identified for each patient.

The DSX Automated ELISA platform by Dynes Technologies with reagents from Inova Diagnostics was used for aCL and aB2GPI determinations. There was no change in methodology during the study period. Cutoffs used were those contained in package inserts. Because the study’s intent was to characterize test-ordering patterns, it did not assess LA, aCL, or aB2GPI interpretation. Data were collected, categorizing results as abnormal (any “positive”); however, chart review would have been necessary for full interpretation of initial test results, reflex testing and confirmatory results. Testing for Lupus Anticoagulant consisted of the dilute Russell’s Viper Venom time and the phospholipid-sensitive (lupus-sensitive) aPTT; if these were abnormal, then reflex testing would be initiated. Investigating the interpretation of LA testing is outside the scope of this study.

The study was conducted in accordance with the ethical principles as set out in the Belmont Report and the Declaration of Helsinki. It was reviewed and approved by the Geisinger Institutional Review Board/Ethics Committee (Approval Number [2021−0630]) with the determination that individual informed consent was not required under the IRB exemption for a quality study, and because it met the criteria for exemption under 45 CFR 46;102(d): a systematic investigation including research development, testing, and evaluation, designed to develop or contribute to generalizable knowledge. Informed consent was therefore not obtained.

Inclusion criteria were defined as outpatients with APS-specific ICD diagnostic codes and/or APS-associated “test collected” codes, between the ages of 18 years of age through 75 years of age, receiving care at Geisinger’s medical facilities at the following service locations of care: Obstetrics/Gynecology (OB/GYN), Neurology, Rheumatology, Emergency Medicine (ED)/Trauma, Gynecology Oncology, Cardiology, Internal Medicine, Cardiothoracic Surgery, and Vascular Surgery. ED/Trauma had been included as a separate service in the dataset to better capture APS testing initiated during an acute event; the data associated with this location was not specific regarding the specialty of the ordering clinician.

The age range was selected based on the mean peak incidence noted in a population in a single center in Minnesota [2]. For both females and males, APS peak incidence has been noted to be consistently associated with age < 75 years and occasionally associated with age > 70 years [1]. An expanded age range would have added data, but would have also added to confounders, based on the increase in age-related thrombotic events in ages > 75 years.

The study data was categorized into 2 data sets to characterize study groups: 1) APS-specific ICD-9 or ICD-10 diagnostic code plus APS-associated test group (APSCT = APS test plus code) and 2) the APS-associated test-only group (ATC = APS test only), which contained patients with APS testing and no APS-specific diagnostic code. The two data sets were filtered for the presence of the following test results: LA, IgG and IgM aCL antibody testing, and IgG and IgM aB2GPI antibody testing, performed at initial contact and then again applied to identify subsequent testing. The time frame for assessing completeness of testing was defined as the specimen collection date, less than or equal to 12 weeks after the first APS sample collection date. Guideline-based confirmatory testing should occur at least 12 weeks after positive testing; therefore, testing collected within 12 weeks of the first test collected (initial testing) was defined as non-confirmatory testing. This definition does not account for any testing performed in the short period before the first date of study data (01/01/2011), or in the short period after the study which could not be captured.

## Results

The original data set consisted of patients with visits in one of the above-noted locations of care within the health system with an APS-associated test alone, an APS-associated ICD-9 or −10 diagnostic code, or an APS-associated ICD diagnostic code with an APS-associated test (n = 25324 screened patients), 78.92% female and 21.08% male (one patient was listed as unknown sex). There were 33 patients with test codes indicating orders for APS testing, but with no test collected (i.e., sample missingness), representing approximately 0.1% of the original sample size of 25324.

Of the screened patients, 8645 had only an APS-associated ICD diagnostic code without an APS test (ACC). Having no testing, the ACC group underwent no further analysis (exclusion depicted in Fig 1); the remaining 16679 patients had either APS testing plus an APS-associated ICD diagnosis code (APSCT) or APS testing alone (ATC). Of 16679, there were 15307 (91.77% of 16679) with only a test (ATC) and 1372 (8.23% of 16,679) with APS testing plus an APS-associated ICD diagnostic code (APSCT).

**Fig 1:**
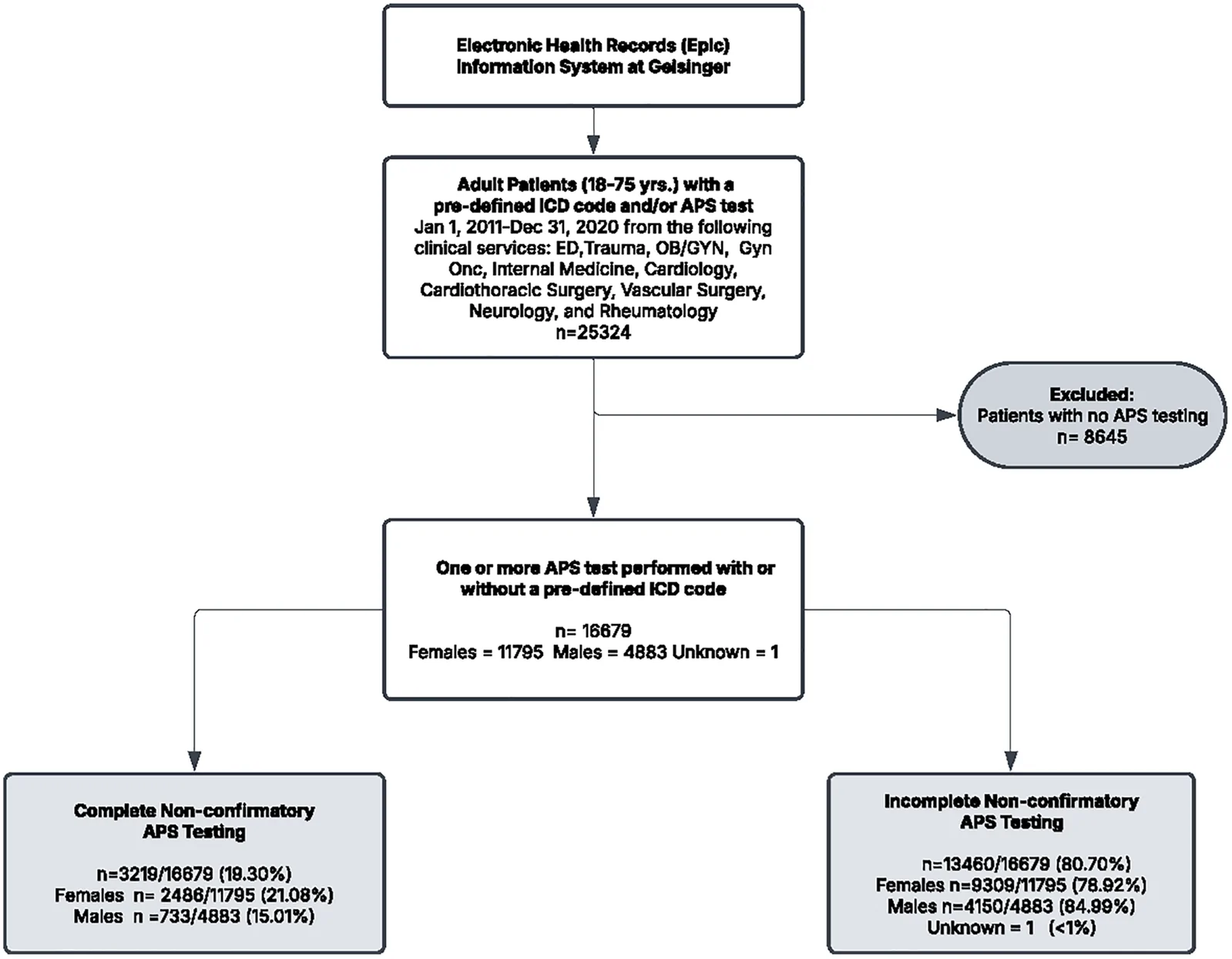
Inclusion and exclusion criteria for study population. The figure outlines the selection process used to extract data and identify participants with APS testing or pre-defined APS-related ICD codes. Criteria included participants aged ≥18-75 yrs. treated in clinical services known to be likely to perform APS testing. Exclusions were defined as patients with no APS-related testing. Since testing for APS is non-confirmatory without additional chart review, the final data sets for comparison were differentiated as “Complete non-confirmatory testing” and “Incomplete non-confirmatory testing”.

Of 1372 patients in the APSCT group (APS-specific diagnostic code plus testing), 81.63% were female, and 18.37% were male. The median age of females in the APSCT group was 32 years, while the median age of males in the APSCT group was 53 years. Of the 15307 patients in the ATC group (testing only), 69.74% were female, and 30.25% were male. The median age of females in the ATC group was 44 years, while the median age of males in the ATC group was 54 years (Fig 2).

**Fig 2:**
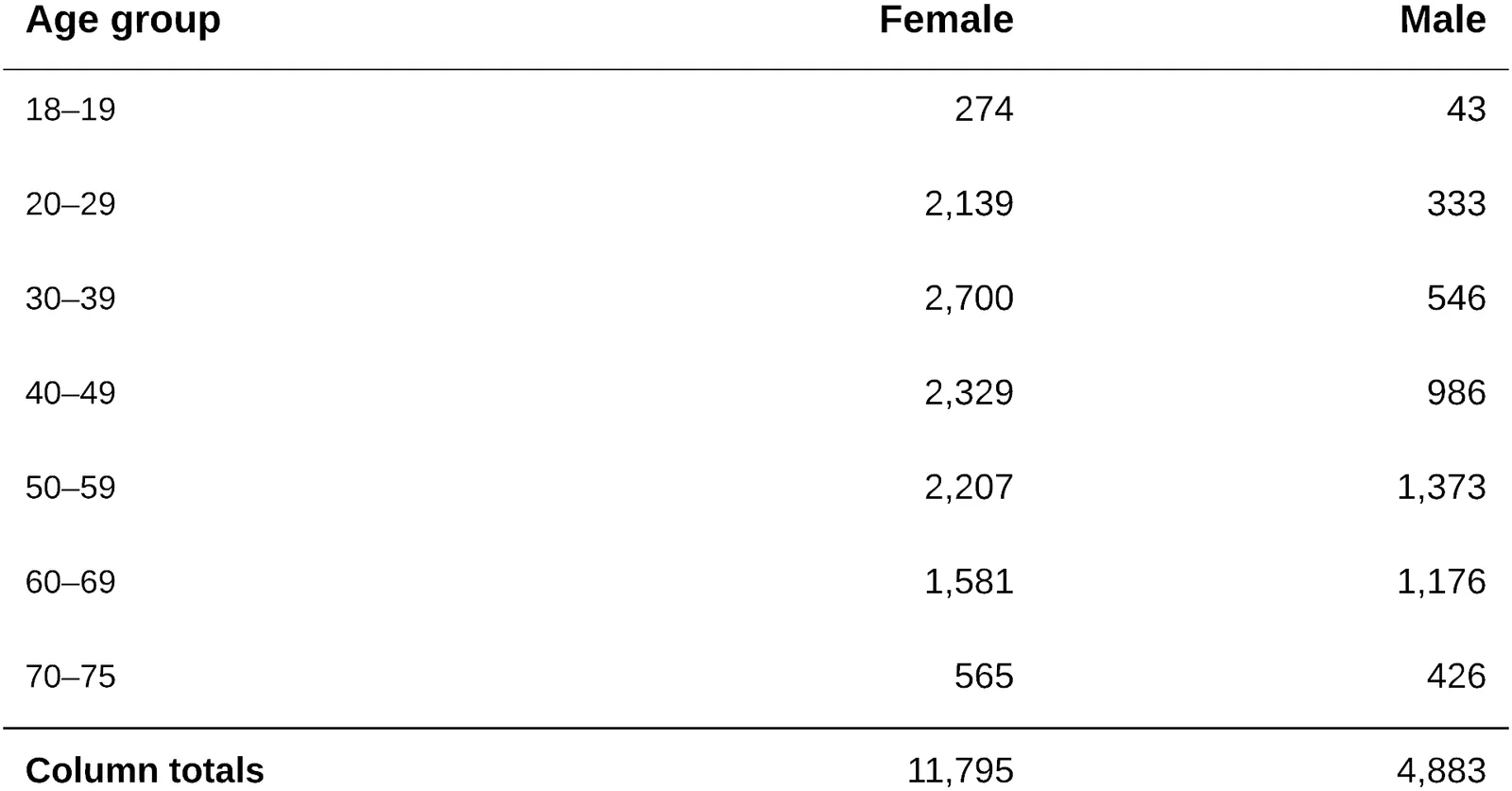
Participants with APS testing. The figure depicts the entire population of participants (n = 16679) who had APS testing ordered, substratified by sex and age. The first age category, 18-19 yrs., and the last age category, 70-75 yrs., do not reflect the 10-year span of the other age categories.

Complete non-confirmatory (initial) APS testing (LA, IgG + IgM aCL antibodies, and IgG + IgM aB2GPI antibodies) was ordered and performed in 3219 patients, representing 19.30% of the 16679 patients with any APS testing ordered. There was a total of 4883 males undergoing APS testing, of which 733 (15.01%) had complete testing. There was a total of 11795 females undergoing APS testing, of whom 2486 (21.08%) had complete testing.

Incomplete non-confirmatory (initial) APS testing was ordered/performed in 13460 (80.70%) of the 16679 patients. The most frequent testing pattern observed in incomplete testing was the performance of only aCL IgG and IgM antibodies. This testing pattern was present in 47.64%, or 6412, of the 13,460 comprising the incomplete initial testing group.

## Discussion

This is a first-of-its-kind study that attempts to characterize the gap in optimal diagnostic testing for APS using longitudinal EHR data. APS testing is important because it is an integral part of the characterization of a rare disease whose manifestations overlap with those of multiple other disease entities. Testing may be ordered by clinicians from many specialties who may be unfamiliar with the current ACR/EULAR criteria for identifying APS. Based on research criteria, Lupus Anticoagulant (LA), IgG and IgM aB2GPI, and IgG and IgM aCL antibody tests should be included in the initial evaluation for APS, and we believe doing so would aid in the clinical diagnosis. The potential utility of using patterns of single-, double- and triple antiphospholipid antibody positivity is recognized, as is the relative impact of isotype on the risk of disease [3,11,12]. Thus, to optimize the use of laboratory testing in the initial evaluation for APS in an appropriate clinical setting, each of these tests should be ordered and performed. This study identified the testing gaps for future investigation of interventions aimed at closing testing gaps.

The central finding of the study is the observation that a large proportion of patients had incomplete non-confirmatory (initial) APS test orders (80.70% of 16679). There was incomplete non-confirmatory testing documented for 9309 of 11795 females (78.92%) and 4150 of 4,883 males (84.99%), and one person of unknown sex. This gap represents an important opportunity to improve testing that could avoid a missed or delayed diagnosis of APS. Slightly more males (84.99%) than females (78.92%) underwent incomplete testing.

Potential reasons for incomplete testing include: provider preference (which was outside the scope of the data extraction); patients screened for autoimmune disease other than for APS alone; clinic encounters from patients new to the health system with referrals or new patients in the health system with follow-up appointments after outside prior evaluation; lack of awareness on the part of the ordering clinician of testing used in the ACR/EULAR criteria; or an interference, such as the presence of an acute thrombosis at the time of testing, requiring a delay in testing (as with the LA test), which could result in loss to follow-up. Further investigation into each potential root cause will be critical to the generalizability of this data.

Another reason for the testing gap could be bias introduced from EHR-based data extraction, causing an inability to differentiate confirmatory testing from initial testing without in-depth chart review, particularly in the setting of initial APS testing being completed in the 12 weeks before the start date of the study, or confirmatory testing in the 12 weeks after the end date. Taken in context, the combined 24 weeks of the total 10 years of data represents only 0.4% of the total in terms of missingness; therefore, missingness would not introduce a large bias.

Likewise, initial (nonconfirmatory) testing conducted at an outside facility should not introduce substantial bias because of Pennsylvania’s Health Information Exchange (KEYHIE), which rapidly integrates with Geisinger data. Finally, the finding that incomplete nonconfirmatory (initial) testing was predominantly comprised of aCL only (47.64%) and/or aB2GPI antibodies could be due to reasons similar to the overall findings.

The study data demonstrated that 91.77% (15307/16679) of initial APS test orders were made without a corresponding diagnostic code, a circumstance which is more likely to indicate initial testing; or represent evaluations for other autoimmune disease (for example, systemic lupus erythematosus). Further studies will be required after interventions to include indications for testing into EHR order sets.

The female component of the APSCT cohorts tended to be younger than the female component of the ATC cohort in this study, suggesting that females are evaluated and diagnosed with APS earlier in life. A female patient population screened for APS in the settings of recurrent pregnancy loss and other pregnancy complications potentially attributable to APS would be younger. However, the age cutoff of 75 years may have slightly biased this finding, as some studies noted a peak in APS in the age group over 75 years [1,2]. The older age of the male component of both ATC and APSCT cohorts is may be due to screening occurring in the setting of a previous acute thrombotic episode, for example, stroke.

Advantages of the study include standardized test options within a unified healthcare system with a standardized EHR and a stable patient population, which made 10 years of longitudinal data available to study providers’ test-ordering patterns for a rare disease. The sample size of this study is independent of the reported low incidence and prevalence of the disease [1,2]. Data collection aimed to evaluate patterns for testing of suspected disease and therefore was not dependent on the presence of disease.

Other advantages include stable testing methods for aCL antibodies, aB2GPI antibodies, and Lupus Anticoagulant, which were available continuously throughout the study. In addition, the Geisinger Phenomic Data Core infrastructure supports mapping local EHR codes to standard terminologies, including ICD-9/10 and LOINC (Logical Observation Identifiers Names and Codes) test codes. In terms of generalizability, the Geisinger catchment area represents a large footprint encompassing both rural and metropolitan settings.

A study limitation, which is common in large EHR-based studies [14], was the lack of incorporation of chart review of the over 25,000 clinical notes. While the study aim was to create a baseline view of testing gaps on which to initiate more specific investigations, the limitation prevented the ability to confirm the identification of testing as initial (non-confirmatory), as opposed to confirmatory testing; the ability to investigate if testing had been initiated prior to the first study date for initiation of data collection; identifying patients being screened for other autoimmune disease (not APS); and review of test ordering patterns by specialty. Anticardiolipin antibody testing, which is a test that can be performed without concern for current or recent anticoagulation, tended to be the first test identified and was considered an indication of initial testing.

Another limitation is the possibility that patients from external hospitals were referred to Geisinger during the study period, potentially causing patients with subsequent APS testing to incorrectly appear to be initial testing; this risk is low given the population stability in the Geisinger population [15]. Finally, the study’s focus on patients age 75 years and younger, was a purposeful limitation, given the presence of thrombotic events in the elderly. The exploration of APS test ordering in the > 75 age group will require future investigation.

## Conclusions

This study suggests that most test ordering practices in the evaluation for APS by selected medical and surgical specialties in this healthcare system are not concordant with the testing described in the ACR/EULAR criteria for the evaluation of antiphospholipid antibody syndrome: anti-Cardiolipin antibody (IgG and IgM); Lupus Anticoagulant; and anti-B2GPI antibody (IgG and IgM). This lack of standardized test ordering practices may represent incomplete laboratory evaluation for the disease. Adequate, accurate test ordering allows efficient disease management (for example, non-thrombotic manifestations may require additional, or a change in, therapies and management) and efficient use of resources. Testing gaps can result in over-and under-diagnosis of APS, leading to delays in appropriate care.

Future interventions such as improved laboratory test menus with test descriptions and EHR nudges are well-positioned to provide guidance for optimal testing practices for antiphospholipid antibody syndrome and to remind physicians of the need to perform complete testing. Addressing patterns of incomplete test ordering can be accomplished by providing information on current guidelines for APS testing and implementing EHR test-ordering indications or order sets, which could help ordering clinicians close the gaps demonstrated here.

## Supporting information

S1 Table Listing of Diagnostic Codes used to query the electronic health system to identify participants who may be subject to testing for antiphospholipid antibody syndrome. This is the list of ICD diagnostic codes used to query the electronic health record system.
(XLSX)
